# Detrimental Industries’ Sponsorship in Football Clubs Across Ten Major Leagues from 2000–2022: A Retrospective Study

**DOI:** 10.3390/nu16213606

**Published:** 2024-10-24

**Authors:** Mikołaj Kamiński, Wiktor Szymajda, Ada Kaczmarek, Matylda Kręgielska-Narożna, Paweł Bogdański

**Affiliations:** 1 Department of the Treatment of Obesity and Metabolic Disorders, and of Clinical Dietetics, Poznań University of Medical Sciences, Szamarzewskiego 82/84, 60-569 Poznan, Poland; matylda-kregielska@wp.pl (M.K.-N.); pawelbogdanski73@gmail.com (P.B.); 2 Student Scientific Club of Clinical Dietetics, Department of the Treatment of Obesity and Metabolic Disorders, and of Clinical Dietetics, Poznań University of Medical Sciences, Szamarzewskiego 82/84, 60-569 Poznan, Poland

**Keywords:** public health, gambling, alcohol, ultra-processed food, advertising, sports

## Abstract

Background: Sponsorship of football teams by detrimental industries may negatively impact fans’ dietary and behavioral choices. The study aimed to determine the proportion of sponsors on the jerseys of teams in the top ten football leagues that comprise companies producing alcohol or unhealthy food, or engaging in gambling. Methods: We conducted a retrospective study, incorporating data from first-division football teams in 10 countries (Argentina, Brazil, England, France, Germany, Italy, Poland, the Netherlands, Spain, and the United States) playing from 2000–2022. Data were collected on the primary sponsors displayed on team jerseys and categorized into alcohol, unhealthy food (defined as producers of ultra-processed food according to the NOVA classification), gambling, or other, based on the nature of the products or services offered by the sponsors. We performed descriptive statistical analyses and multivariate linear regression analyses. Results: A total of 4452 sponsorship records were analyzed. The majority were classified as “other” (81.8%), followed by gambling (6.9%), alcohol (2.6%), and unhealthy food (2.6%). We did not identify any sponsor representing the tobacco industry. The prevalence of gambling sponsors surged from 1.7% in 2000 to 16.3% in 2022. Conversely, alcohol-related sponsorships dwindled from 6.2% in 2000 to 1.0% in 2022. In the multivariate linear regression model, these trends were statistically significant. The alcohol industry remained visible in the Spanish league. Conclusions: A significant proportion of sponsorships on the jerseys of top football teams across the world represents alcohol, ultra-processed food, or the gambling industry. Trends in the types of sponsors on the jerseys of leading football clubs across the Western world are diverse. Particularly concerning is the recent increase in the percentage of clubs sponsored by the gambling industry. To limit the detrimental effects of the promotion of unhealthy products, novel policies should be considered.

## 1. Introduction

Detrimental industries, such as the producers of alcohol or tobacco and companies offering gambling services, are often subject to numerous legal regulations aimed at restricting the marketing activities of these firms. As a result, companies are altering their marketing strategies, often seeking opportunities for indirect advertising of their products, as well as ways to foster a positive image among consumers.

Although detailed data on marketing expenditures by detrimental industries is scarce, it appears that in the alcohol industry, companies spend anywhere from several to tens of percents of their total expenditures on various forms of advertising each year [1]. Similarly, in the case of the gambling industry, it is estimated that marketing expenditures are increasing year by year, with companies actively engaging in the sports sector [2]. In the case of a heavily regulated industry, such as tobacco, it is estimated that while direct advertising expenditures have decreased, indirect marketing expenditures have increased [3,4]. In comparison to the alcohol, tobacco, and gambling industries, manufacturers of ultra-processed food face minimal restrictions on advertising their products. As a result, in certain areas, advertisements for ultra-processed food dominate over other products [5,6], which is one of the main factors contributing to poor dietary decisions [7,8].

Football is one of the most popular sports around the world, drawing the attention of billions of people [9]. Sponsorship of football teams by food and beverage companies may have a considerable negative impact on the dietary choices of fans [10]. The pervasive marketing and advertising strategies employed by these sponsors often promote energy-dense, nutrient-poor products, which contribute to unhealthy eating habits and the rising prevalence of obesity and non-communicable diseases [11]. For example, Kelly et al. found a relationship between exposure to alcohol-branded sports sponsorship and increased alcohol consumption among young Australians in a cross-sectional survey study [12]. Bestman et al. demonstrated that school-aged children who are interested in sports can easily recall and recognize the sponsor of a sports club [13]. Therefore, marketing through sponsorships of popular sport clubs affects not only adult fans but may also influence children’s consumption of products like ultra-processed food.

The association between football teams and their sponsors may lead to increased brand exposure and loyalty among fans, as they may perceive the endorsed products as being linked to the success and appeal of their favorite teams. This phenomenon, known as the “halo effect”, can create a biased perception of the nutritional quality of the sponsored products, ultimately influencing fans to consume unhealthy food and beverages more frequently [14,15]. The halo effect is also defined as “a common bias, in the impression people form of others, by which attributes are often generalized” [16], which in practice means that a well-presented product is automatically perceived as good, even though its properties are not precisely known. Therefore, football club fans, even if they are not familiar with the products of a specific sponsor, may still be influenced by the halo effect, as the sponsor’s ubiquitous logo becomes associated with the authority of their beloved team. As a result, they may be more inclined to seek out the sponsor’s products or services. Consequently, it is crucial to critically evaluate and potentially regulate the nature of sponsorships within the realm of sports, promoting healthier food choices and fostering a more responsible marketing environment.

There is a growing need to analyze the nature and impact of sponsorships in football teams to better understand their potential influence on public health and consumer behavior [17,18,19,20,21,22,23,24]. However, previous studies analyzed a limited number of leagues, seasons, and teams, which limits the impact of the results. A comprehensive analysis of the sponsors on the jerseys of football teams may detect areas for intervention and policy development to limit the promotion of unhealthy products [10,25].

The study aimed to determine the proportion of sponsors on the jerseys of teams in the top 10 football leagues that comprise companies producing alcohol or unhealthy food, or engaging in gambling.

## 2. Materials and Methods

A study identifying the sponsorship of football teams through publicly available internet sources does not require ethical committee approval, as it involves the collection of information from publicly accessible platforms and does not involve direct interaction with human subjects. Moreover, the data collected in this manner are not of a sensitive nature and pose no risk to the privacy or well-being of individuals. Consequently, such research falls outside the scope of ethical considerations typically required for studies involving human participants.

The study is retrospective in nature. It included football teams from the first divisions of the following ten countries: Argentina (Primera División), Brazil (Serie A), England (Premier League), France (Ligue 1), Germany (Bundesliga), Italy (Serie A), Poland (Ekstraklasa), the Netherlands (Eredivisie), Spain (Primera División), and the United States (Major League Soccer). The study covered the seasons from 2000 (2000/2001) to 2022 (2022/2023). For simplification purposes, if the entire season took place within a single year, for example, 2000, or commenced in the latter half of 2000 (2000/2001), it was considered to represent the year 2000. This convention was applied consistently in subsequent years. Importantly, in Argentina, in 2020, football games were canceled due to the COVID-19 pandemic [26].

For each season and team, information was collected on the name of the main sponsor displayed on team football jerseys and the products or services offered by the sponsor. We sought data on the sponsors featured on the jerseys of specific teams using open internet sources. Initially, the authors involved in this process (W.S. and A.K.) searched the official websites of sports clubs and football leagues, general information sites such as Wikipedia, as well as the website “Football Kit Archive” (https://www.footballkitarchive.com, accessed on January 2023 to May 2023), which collects data on jerseys. In situations where we could not identify the sponsor on a club’s jersey for a given season, we entered the club’s name, year, and the phrases “jersey”, “logo”, or “sponsor” into the Google search engine. We searched websites and images. Additionally, if we were seeking information, for example, about a Dutch club, these queries were translated into the local language using Google Translate. If neither of the two authors involved in identifying the sponsor on the football club’s jersey for a given season could identify such a sponsor, it was deemed that no sponsor was present. After collecting the data, a classification of each type of offered product and service was conducted as follows: (a) alcohol for companies involved in the production or distribution of alcohol, for example, beer; (b) unhealthy food for companies producing ultra-processed food according to the NOVA classification, such as sugary beverages, cookies, salty snacks, etc. [27] (the term “ultra-processed food” in the text refers to the category of “unhealthy food”); and (c) gambling, including bookmakers, online casinos, and land-based casinos. The remaining products and services were classified as “others”, and cases where no sponsor was displayed on the jersey were classified as “no sponsor”. Initially, we included a category for “tobacco products” but did not find any records matching this category. The classification was carried out by W.S. and A.K., after which M.K. independently conducted the classification. In cases of doubt, the three co-authors jointly determined the classification.

The data used in this study are attached to the paper as Supplementary File S1. Data processing, statistical calculations, and visualizations were conducted using the R programming language (version 4.4.1, R Foundation, Vienna, Austria). The data were subjected to descriptive statistics. Additionally, we performed a multivariate linear regression analysis to assess the temporal trends in the proportion of various sponsor categories over multiple seasons. The independent variable used in each regression model was each consecutive season (coded from 1 to 23), representing the passage of time. The dependent variables were the percentage shares of sponsor categories. Each category was analyzed individually to determine whether its proportion exhibited a significant increase or decrease across the seasons. The analysis allowed us to assess both the general trend (positive estimates represent increases in the share of a specific sponsor category over time, while negative estimates represents a negative trend) and the statistical significance of changes over time. *p*-values < 0.05 were considered statistically significant.

## 3. Results

Overall, we collected n = 4452 records, characterizing the sponsors on the jerseys of teams in the top 10 football leagues in 23 consecutive seasons. Most of the sponsors were classified as “other” (n = 3641; 81.8%), followed by “gambling” (n = 307; 6.9%), “alcohol” (n = 117; 2.6%), and “unhealthy food” (n = 115; 2.6%). In n = 272 cases, the team did not have a sponsor on their jerseys.

**Figure 1:**
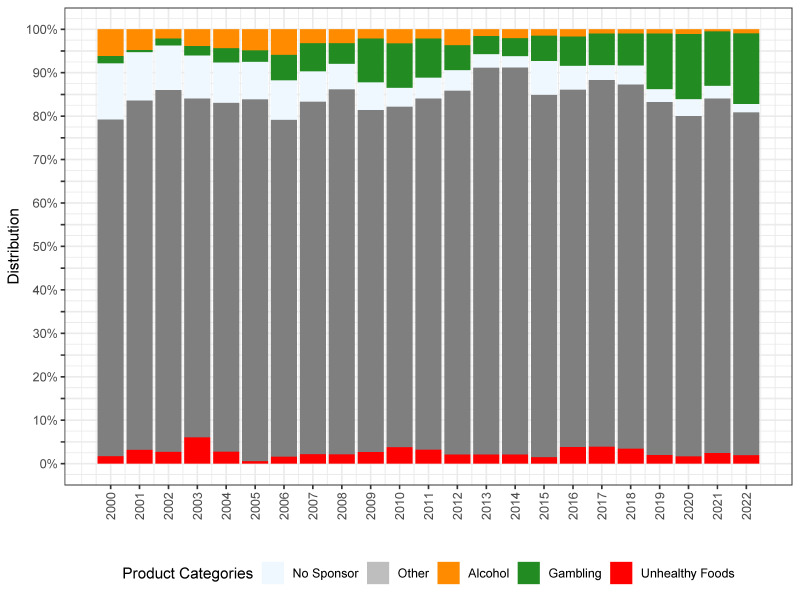
Distribution of different types of sponsors on the jerseys of football teams of ten analyzed leagues.

To facilitate the analysis of trends, we created a visualization of our data. Figure 1 aggregates statistics for all leagues over the analyzed years. We found that the frequency of sponsors related to gambling increased from 1.7% in 2000 to 16.3% in 2022 (Figure 1). The proportion of the alcohol industry among sponsors on the jerseys of teams decreased from 6.2% in 2000 to 1.0% in 2022. The percentage of sponsors representing unhealthy food fluctuated between 0.5% to 6.0% in the analyzed period. These trends were confirmed in the multivariate linear regression model (Table 1). We found that, over the seasons analyzed across the ten leagues, the percentage of sponsors offering alcoholic beverages significantly decreased, while the percentage of those offering gambling services increased. No significant trends were observed regarding manufacturers of unhealthy food or in the “other” category.

**Figure 2:**
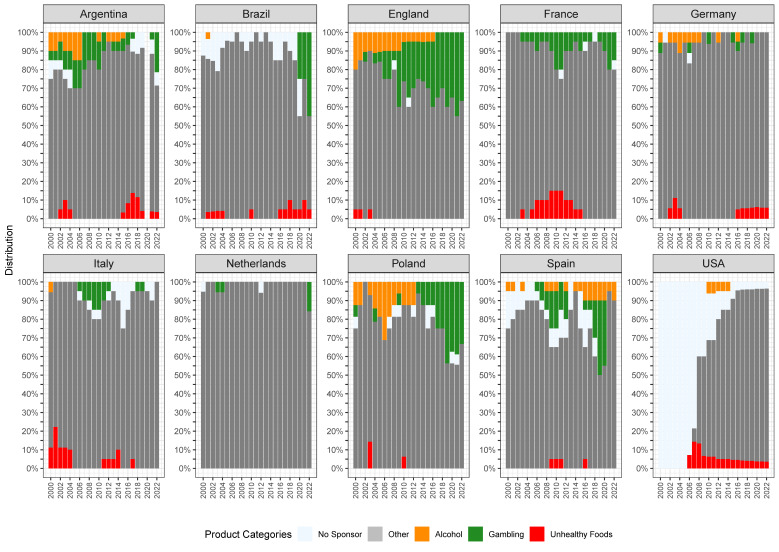
Distribution of different types of sponsors on the jerseys of football teams across ten major leagues.

**Figure 3:**
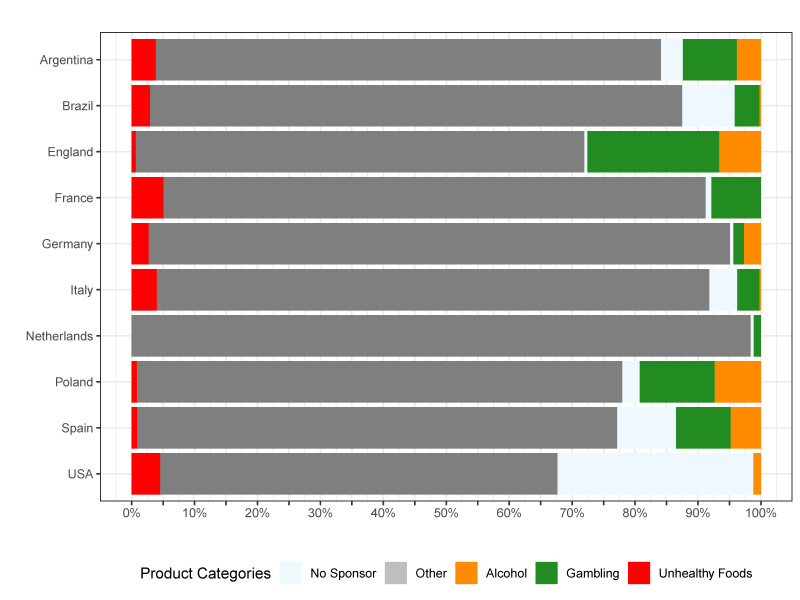
Cumulative distribution of different types of sponsors on the jerseys of football teams across ten major leagues from 2000–2022.

Figure 2 illustrates the distribution of various categories of sponsors over the analyzed seasons, broken down by the ten football leagues. Particularly noteworthy is the significant variation in the trends of sponsor categories across the different leagues (Figure 2). The percentage of companies offering gambling services has increased in the last years in Brazil, England, Poland, and, to some extent, France (Figure 2). The alcohol industry nearly disappeared from jerseys in the analyzed leagues over time, except in Spain. The sponsors representing unhealthy food continue to appear on the jerseys of football teams in Argentina, Brazil, Germany, and the USA. Figure 3 presents the cumulative percentage of all categories of sponsors over the analyzed seasons. Among all of the analyzed leagues, the Eredivisie from the Netherlands had the lowest proportion of detrimental industry presence on their jerseys (Figure 2 and Figure 3).

**Figure:**
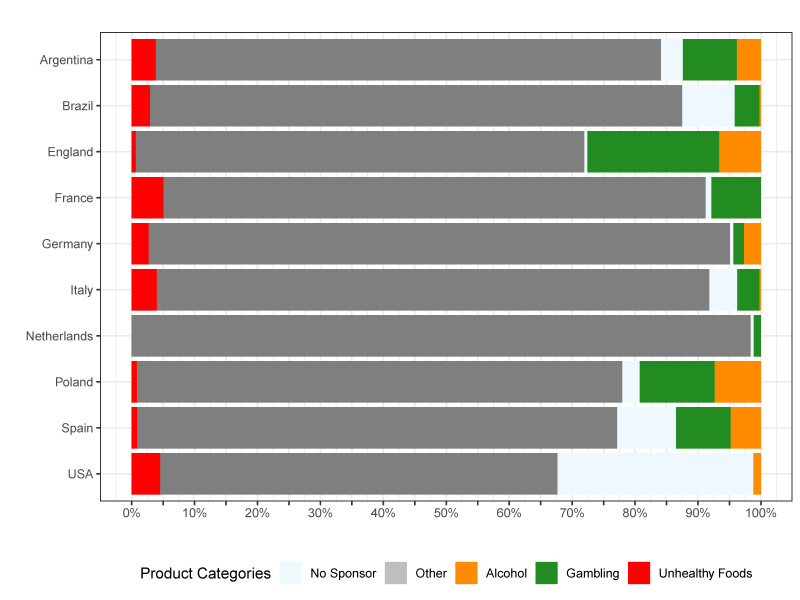


## 4. Discussion

In our study, we characterized the sponsors on the jerseys of football clubs in the top 10 important football leagues over the course of 23 consecutive seasons. We demonstrated that a significant proportion of sponsors were associated with the alcohol industry, ultra-processed food, and gambling.

### 4.1. Main Findings

Among the analyzed types of sponsors, companies associated with gambling were most prominently featured on the jerseys of football teams across the examined leagues. A significant prevalence of gambling-related sponsors has been particularly noted in recent years in Brazil, England, and Poland, where they represented over 40% of the clubs’ sponsorships. However, a shift is occurring; clubs in the English Premier League have decided to cease featuring the names and logos of gambling firms on their jerseys starting from the 2026/2027 season [28]. In Italy, a government prohibition on showcasing gambling sponsors on football teams’ jerseys has been in effect since 2019 [29], with Spain implementing a similar ban in the 2021/2022 season [29,30] (Table 2).

While the promotion of gambling is not directly associated with health hazards, it is essential to underscore that it can lead to fans making risky and financially detrimental decisions. This is especially concerning for vulnerable individuals who could develop life-ruining addictions [31]. The threat is particularly pronounced among young people who, having grown up attached to their favorite clubs, are accustomed to a positive image of companies offering gambling services [32].

In recent years, many countries have noted the near disappearance of alcohol industry sponsorships from football jerseys. In France, there is a complete prohibition on sponsorships by the alcohol industry [33]. In Poland, any advertising of alcoholic beverages except for beer is prohibited [34]. In the sole country where sponsorships associated with the alcohol industry are still observed on football jerseys, namely, Spain, the advertisement of alcoholic products is regulated at the regional level [35]. Previous studies have highlighted the significant frequency of alcohol industry sponsorships among sports teams [18,21]. However, current regulations have led to a decline in the presence of alcohol-related sponsorships on football jerseys.

In the examined countries, only a few percent of sponsors were associated with offering ultra-processed food. The percentage of sponsors on football jerseys associated with ultra-processed food has not changed significantly over the analyzed seasons. However, depending on the country, these trends varied; often for a given league, the percentage of sponsors remained relatively constant from year to year, as seen in Brazil, France, Germany, and the USA. Importantly, producers of unhealthy food were the least regulated group of sponsors analyzed in our study. In 2019, England introduced a restriction for Premier League clubs, prohibiting the advertisement of products high in fat, sugar, or salt on replica football jerseys intended for children [36,37]. While the regulation introduced in England appears to be appropriate, it remains uncertain whether it will significantly impact children’s health. Primarily, the advertising of ultra-processed food in connection with sports is only a small part of the extensive marketing efforts exerted by the food industry on consumers. Therefore, advertising within the sports sphere may prove insufficient in addressing the broader influence of this industry. Furthermore, such regulations may fail to achieve their intended objectives, as companies will adjust their product selection or modify their formulations to meet the criteria set by legal authorities, depending on the strictness of the regulations.

To the best of our knowledge, no regulations concerning the presence of industries related to alcohol, unhealthy food, or gambling have been introduced in Argentina, Brazil, or Germany.

Significantly, in our study, we did not observe any instances where the jerseys of the analyzed leading football clubs displayed logos of companies engaged in producing tobacco products. This is a result of the regulations in effect in the countries under study [38,39,40].

### 4.2. Strengths and Practical Implications

This study’s strengths lie in its extensive analysis of sponsorships across the top ten football leagues over 23 seasons, offering valuable insights into evolving trends in the sector. The categorization of sponsors provides a clear picture of the prevalence of alcohol, unhealthy food, and gambling sponsorships, laying a groundwork for policy development and public awareness initiatives.

The practical implications are significant, providing policymakers with data to develop regulations that balance commercial interests with public health concerns. The study also serves to raise public awareness, enabling fans and consumers to make informed decisions regarding their dietary and behavioral choices influenced by sports sponsorships.

Our results indicate that some football teams are sponsored by producers of alcohol, ultra-processed food, or gambling services. However, based solely on our cross-sectional study, no conclusions can be drawn regarding whether fans’ exposure to sponsors’ logos and names actually translates into consumption decisions. Consequently, further research is needed on this topic, including examining both the individual perspective of football fans and their personal perceptions, as well as their recall and association of the sponsors of their favorite clubs with their choices. Additionally, novel studies are required to analyze how consumption of products advertised on jerseys by the team’s main sponsor changes within a given region inhabited by the fans of that team during the sponsorship period.

There is also the question of what the long-term effects are of fans’ exposure to advertising from detrimental industries on the jerseys of their favorite clubs. It is likely that the longer such a company sponsors a given club, the more fans may identify with that company and its products. We suspect that there is also a cross-sectional effect. This means that if there are many sponsors from the same category, such as those offering gambling services, in a given league, it may influence the fans of that league through increased exposure to such advertisements. Nevertheless, to the best of our knowledge, there are no studies assessing the long-term effects of sponsorship by detrimental industries in sports. Therefore, this issue also requires further research.

### 4.3. Limitations

The study has several limitations. The focus on the top ten football leagues limits the findings’ applicability to other leagues and sports. Our classification of the sponsor might also be perceived as a limitation of the study. We preferred to keep the classification as simple as possible to facilitate the interpretation of the results. An overly detailed classification would have resulted in excessively dispersed outcomes that could complicate interpretation. However, in the Supplementary Materials, we included the dataset with names of the sponsor along with the category assigned to each sponsor. Thus, our entire classification can be verified. Furthermore, the study lacks qualitative insights into the impact of sponsorships on fans’ behavior. Additionally, other influential factors, like advertising intensity and logo placement, are not considered, potentially affecting the comprehensiveness of the results. Although the name and logo of the main sponsor of a football club are most commonly displayed on the jerseys, we must also consider that the name of the football stadium may include the name of another sponsor, for example. Additionally, advertisements for other companies are displayed on banners around the pitch. Individual players may also sign personal contracts and promote companies offering alcohol, unhealthy food, and gambling services. Finally, many of the analyzed clubs have global fame, and their fanbase often extends beyond the region where the stadium is located, with a significant following dispersed around the world. For this reason, analyzing how sponsorship of a football club by a particular company influences the consumer choices of that club’s fans is highly nuanced and requires further research.

## 5. Conclusions

A significant proportion of sponsorships on the jerseys of top football teams across the world represents alcohol, ultra-processed food, or the gambling industry. Trends in the types of sponsors on the jerseys of leading football clubs across the Western world are diverse. Particularly concerning is the recent increase in the percentage of clubs sponsored by the gambling industry. To limit the detrimental effects of the promotion of unhealthy products, novel policies should be considered.

## Figures and Tables

**Table 1:** Results of the multivariate linear regression model. The dependent variables were the percentage shares of sponsor categories among all of the analyzed leagues.

Sponsor’s Category	Independent Variable	Estimate	Standard Error	t Statistics	*p*-Value
Alcohol	(Intercept)	5.20	0.39	13.3	<0.001
	Season	−0.209	0.09	−7.3	<0.001
Gambling	(Intercept)	0.568	1.13	0.5	0.62
	Season	0.520	0.08	6.3	<0.001
Unhealthy food	(Intercept)	2.77	0.50	5.6	<0.001
	Season	−0.0164	0.04	−0.5	0.66
Others	(Intercept)	80.4	1.39	57.8	<0.001
	Season	0.112	0.10	1.1	0.28
No sponsor	(Intercept)	11.1	0.63	17.5	<0.001
	Season	−0.407	0.05	−8.8	<0.001

**Table 2:** Regulations of sponsorship of football clubs in the analyzed countries.

Country	Alcohol	Gambling	Unhealthy Food
Argentina	none	none	none
Brazil	none	none	none
England	none	Premier League clubs have agreed to withdraw gambling sponsorships from the front of clubs’ matchday shirts starting from the 2026/2027 season. https://www.premierleague.com/news/3147426, accessed on 11 July 2023	In 2019, the English Premier League introduced restrictions on the advertising of HFSS (high-fat, high-sugar, or high-salt) products on children’s replica shirts. https://foodactive.org.uk/wp-content/uploads/2021/11/Kicking-Out-Junk-Food.pdf, accessed on 17 July 2023 The UK Code of Non-broadcast Advertising and Direct & Promotional Marketing (CAP Code), Rule 15.18
France	Sponsorship is forbidden in all areas. French Public Health Code art. L. 3323-2	none	none
Germany	none	none	none
Italy	none	Gambling-related football sponsorships have been banned by the government since 2019. https://theathletic.com/4383423/2023/04/07/premier-league-betting-italy-spain/, accessed on 11 July 2023 Article-9 of the Dignity Decree (2018)	none
Netherlands	none	The government-proposed ban on gambling advertising comes into effect in January 2025, but the Football Association opposes the decision. https://www.sportcal.com/betting/sports-betting-advertising-restrictions-planned-in-netherlands/, accessed on 11 July 2023	none
Poland	Ban on advertising and promotion of any alcohol beverage, except for beer. Ustawa o wychowaniu w trzeźwości i przeciwdziałaniu alkoholizmowi, Dz.U. z 2023 r. poz. 165, art. 13	Betting companies that are legally registered and have permission from the Ministry of Finance are allowed to sponsor. Ustawa hazardowa, Dz.U. z 2023 r. poz. 227, art. 29b	none
Spain	There is no state-level legislation in Spain covering alcohol marketing. The advertising of alcoholic beverages is regulated at the regional level. EUCAM (2022) Regulations on Alcohol Marketing. Available at: https://eucam.info/regulations-on-alcohol-marketing, accessed on 13 July 2023	Gambling-related football sponsorships have been banned by the government since the 2021/2022 season. https://ecija.com/en/sala-de-prensa/royal-decree-958-2020-of-3-november-on-commercial-communications-of-the-gambling-activities/, accessed on 13 July 2023 Royal Decree on the Commercial Communications of Gambling Activities, Royal Decree 958/2020. Art. 1	none
The United States	No restrictions for beer and wine. Liquor drinks marketing has been allowed since 2019. Under 21s are excluded from any form of alcohol-related marketing or such sponsors’ appearance on their club uniforms. https://www.mlssoccer.com/news/mls-opens-commercial-sponsorships-sports-betting-spirits-categories, accessed on 14 July 2023 MLS Commercial Sponsorship Guidelines, 26 June 2019.	Sports betting companies have been allowed on shirts since 2020. https://www.mlssoccer.com/news/mls-opens-commercial-sponsorships-sports-betting-spirits-categories MLS Commercial Sponsorship Guidelines, 26 June 2019.	none

## Data Availability

The original contributions presented in the study are included in the article, further inquiries can be directed to the corresponding author.

## Supplementary Materials

The following are available online at https://www.mdpi.com/article/10.3390/nu16213606/s1, File S1: The dataset utilized in the study.
